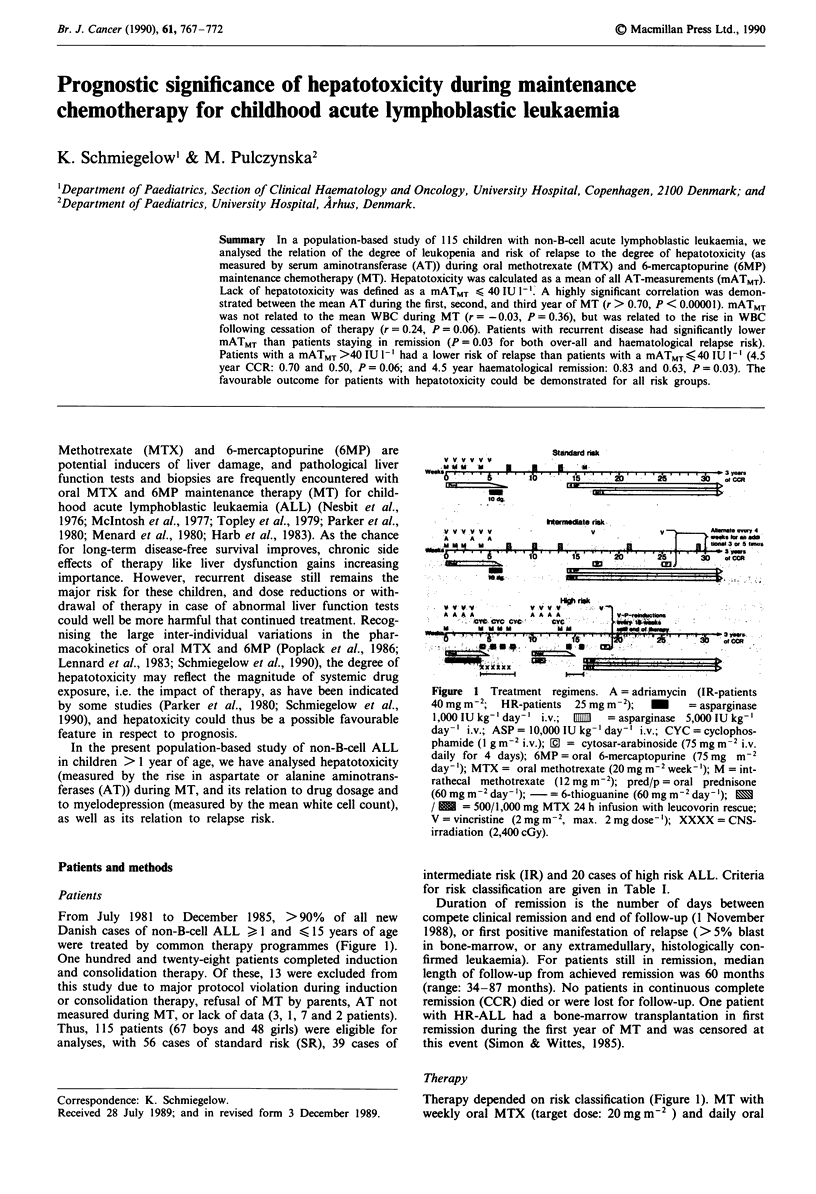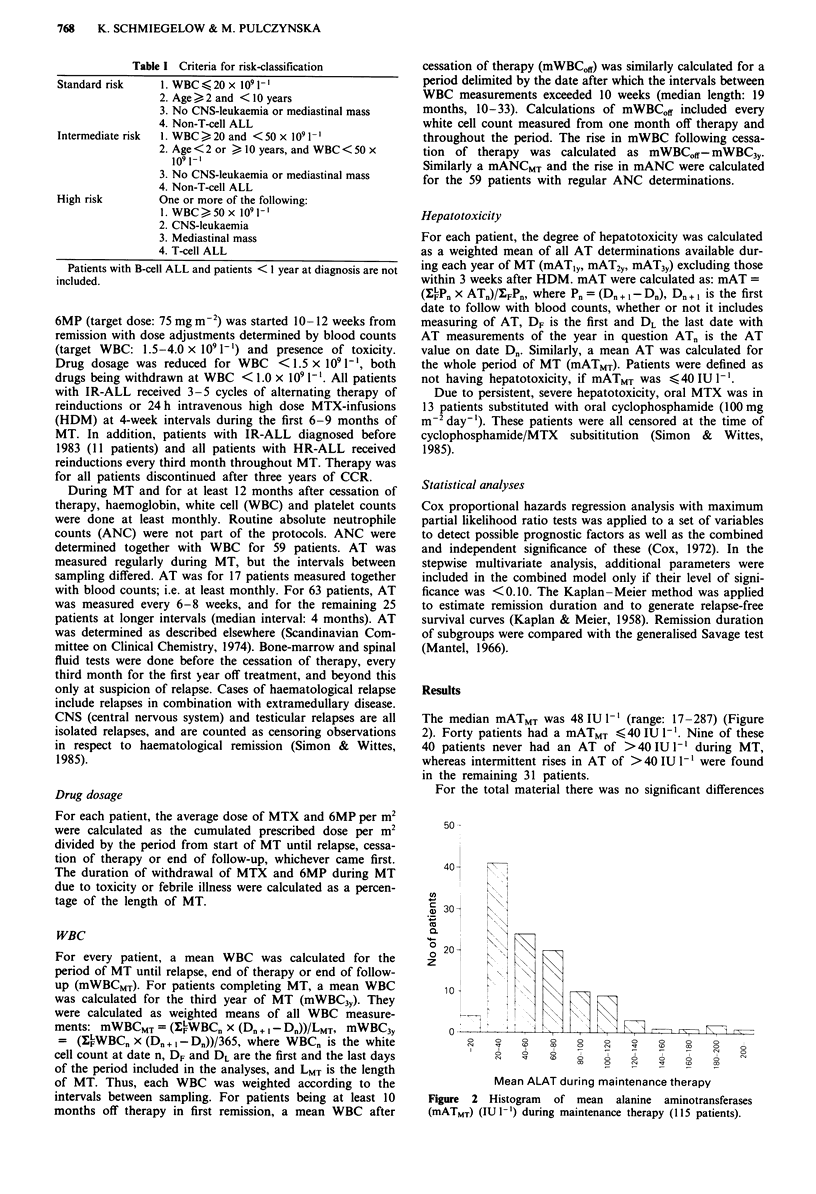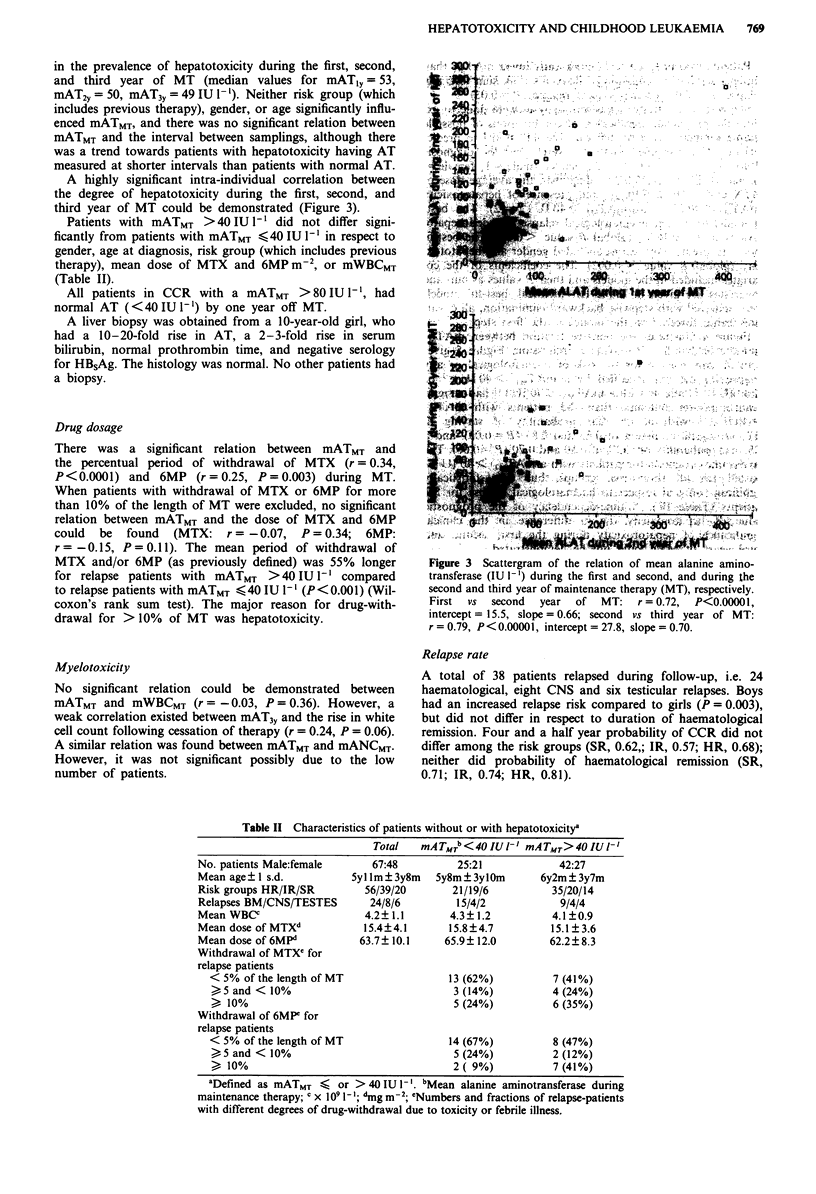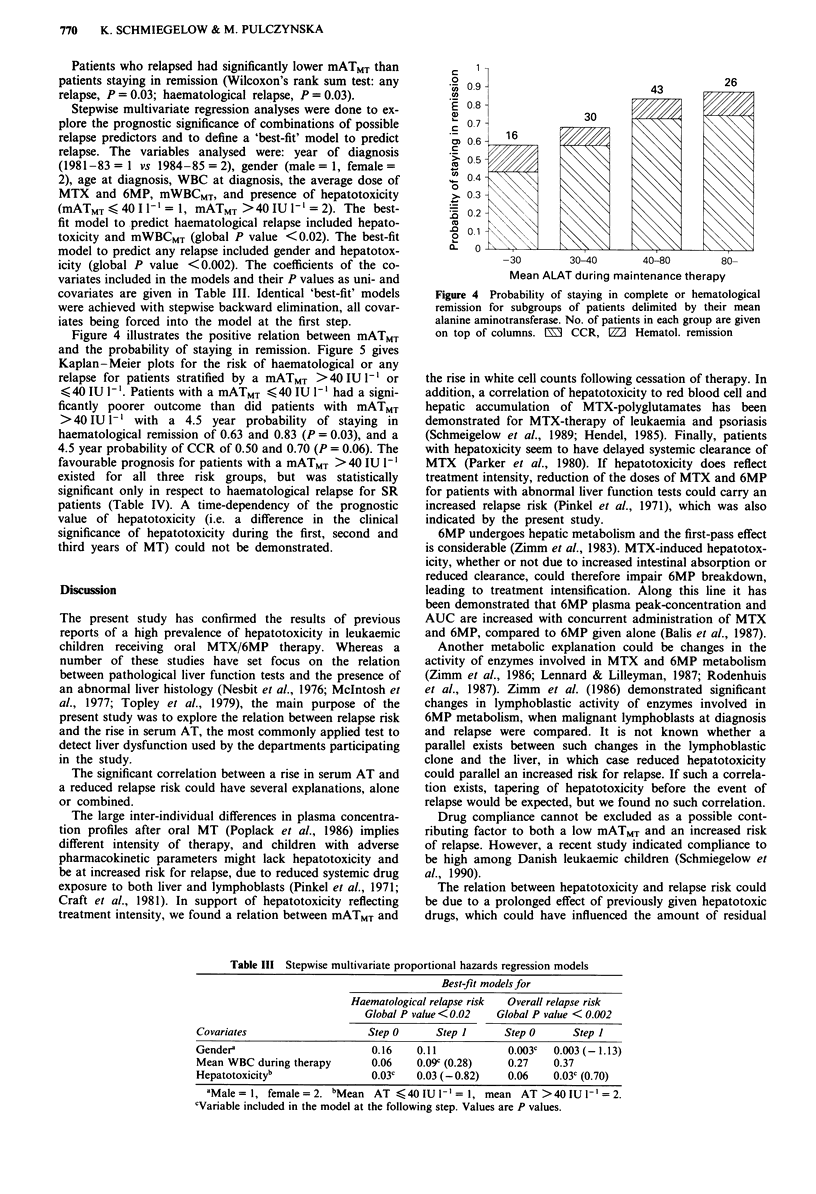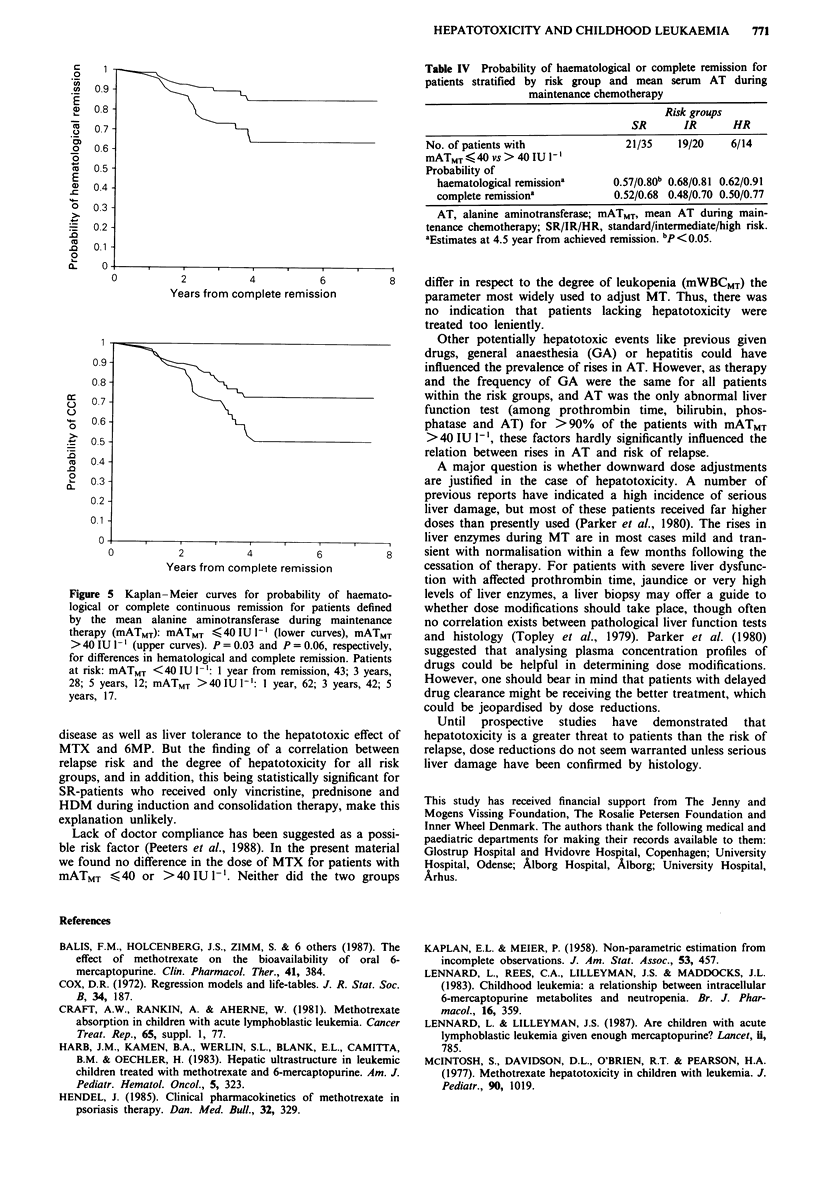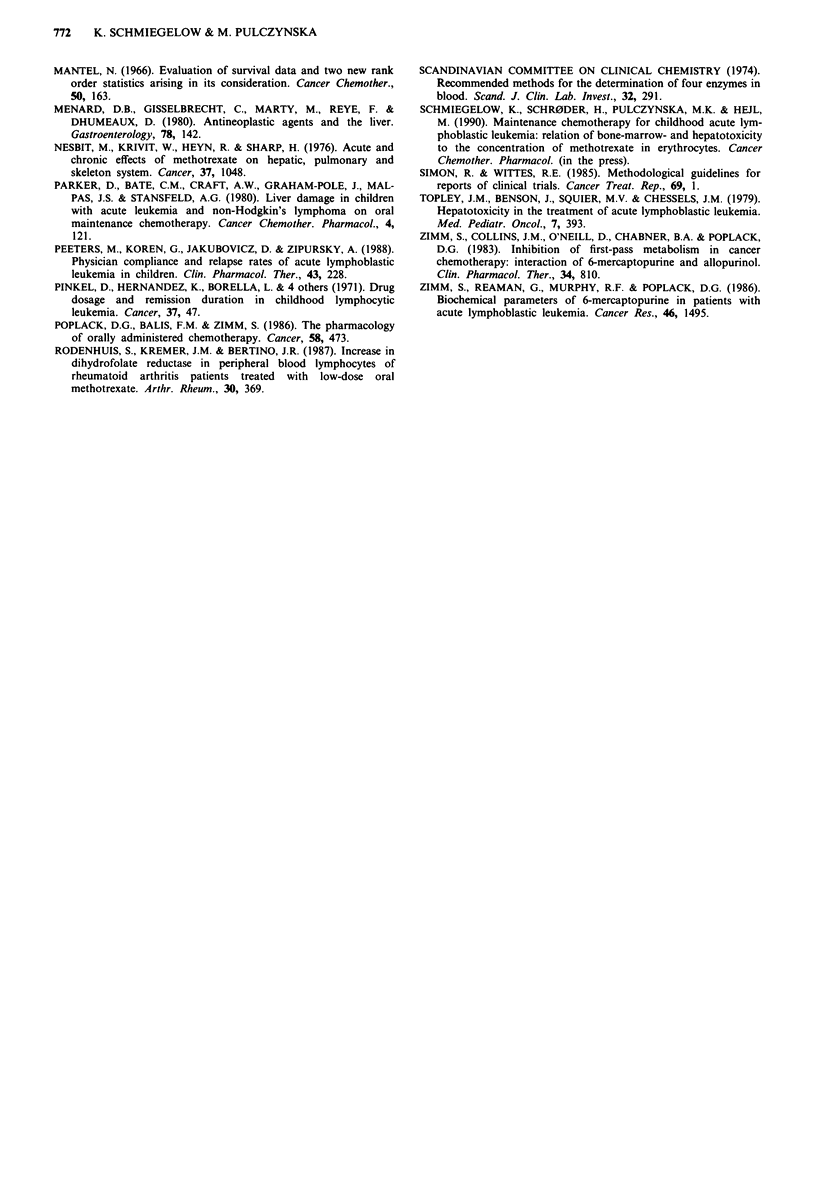# Prognostic significance of hepatotoxicity during maintenance chemotherapy for childhood acute lymphoblastic leukaemia.

**DOI:** 10.1038/bjc.1990.172

**Published:** 1990-05

**Authors:** K. Schmiegelow, M. Pulczynska

**Affiliations:** Department of Paediatrics, University Hospital, Copenhagen, Denmark.

## Abstract

In a population-based study of 115 children with non-B-cell acute lymphoblastic leukaemia, we analysed the relation of the degree of leukopenia and risk of relapse to the degree of hepatotoxicity (as measured by serum aminotransferase (AT] during oral methotrexate (MTX) and 6-mercaptopurine (6MP) maintenance chemotherapy (MT). Hepatotoxicity was calculated as a mean of all AT-measurements (mATMT). Lack of hepatotoxicity was defined as a mATMT less than or equal to 40 IUl-1. A highly significant correlation was demonstrated between the mean AT during the first, second, and third year of MT (r greater than 0.70, P less than 0.00001). mATMT was not related to the mean WBC during MT (r = -0.03, P = 0.36), but was related to the rise in WBC following cessation of therapy (r = 0.24, P = 0.06). Patients with recurrent disease had significantly lower mATMT than patients staying in remission (P = 0.03 for both over-all and haematological relapse risk). Patients with a mATMT greater than 40IUl-1 had a lower risk of relapse than patients with a mATMT less than or equal to 40IUl-1 (4.5 year CCR: 0.70 and 0.50, P = 0.06; and 4.5 year haematological remission: 0.83 and 0.63, P = 0.03). The favourable outcome for patients with hepatoxicity could be demonstrated for all risk groups.


*Br. J. Cancer* (1990), **61**, 767–772 © Macmillan Press Ltd., 1990

### Prognostic significance of hepatotoxicity during maintenance chemotherapy for childhood acute lymphoblastic leukaemia

K. Schmiegelow[1] & M. Pulczynska[2]

[1]*Department of Paediatrics, Section of Clinical Haematology and Oncology, University Hospital, Copenhagen, 2100 Denmark; and* [2]*Department of Paediatrics, University Hospital, Århus, Denmark.*

**Summary** In a population-based study of 115 children with non-B-cell acute lymphoblastic leukaemia, we analysed the relation of the degree of leukopenia and risk of relapse to the degree of hepatotoxicity (as measured by serum aminotransferase (AT)) during oral methotrexate (MTX) and 6-mercaptopurine (6MP) maintenance chemotherapy (MT). Hepatotoxicity was calculated as a mean of all AT-measurements ($mAT_{MT}$). Lack of hepatotoxicity was defined as a $mAT_{MT} \leqslant 40\,IU\,l^{-1}$. A highly significant correlation was demonstrated between the mean AT during the first, second, and third year of MT ($r > 0.70$, $P < 0.00001$). $mAT_{MT}$ was not related to the mean WBC during MT ($r = -0.03$, $P = 0.36$), but was related to the rise in WBC following cessation of therapy ($r = 0.24$, $P = 0.06$). Patients with recurrent disease had significantly lower $mAT_{MT}$ than patients staying in remission ($P = 0.03$ for both over-all and haematological relapse risk). Patients with a $mAT_{MT} > 40\,IU\,l^{-1}$ had a lower risk of relapse than patients with a $mAT_{MT} \leqslant 40\,IU\,l^{-1}$ (4.5 year CCR: 0.70 and 0.50, $P = 0.06$; and 4.5 year haematological remission: 0.83 and 0.63, $P = 0.03$). The favourable outcome for patients with hepatotoxicity could be demonstrated for all risk groups.

Methotrexate (MTX) and 6-mercaptopurine (6MP) are potential inducers of liver damage, and pathological liver function tests and biopsies are frequently encountered with oral MTX and 6MP maintenance therapy (MT) for childhood acute lymphoblastic leukaemia (ALL) (Nesbit *et al.*, 1976; McIntosh *et al.*, 1977; Topley *et al.*, 1979; Parker *et al.*, 1980; Menard *et al.*, 1980; Harb *et al.*, 1983). As the chance for long-term disease-free survival improves, chronic side effects of therapy like liver dysfunction gains increasing importance. However, recurrent disease still remains the major risk for these children, and dose reductions or withdrawal of therapy in case of abnormal liver function tests could well be more harmful that continued treatment. Recognising the large inter-individual variations in the pharmacokinetics of oral MTX and 6MP (Poplack *et al.*, 1986; Lennard *et al.*, 1983; Schmiegelow *et al.*, 1990), the degree of hepatotoxicity may reflect the magnitude of systemic drug exposure, i.e. the impact of therapy, as have been indicated by some studies (Parker *et al.*, 1980; Schmiegelow *et al.*, 1990), and hepatoxicity could thus be a possible favourable feature in respect to prognosis.

In the present population-based study of non-B-cell ALL in children >1 year of age, we have analysed hepatotoxicity (measured by the rise in aspartate or alanine aminotransferases (AT)) during MT, and its relation to drug dosage and to myelodepression (measured by the mean white cell count), as well as its relation to relapse risk.

**Figure 1** Treatment regimens. A = adriamycin (IR-patients $40\,mg\,m^{-2}$; HR-patients $25\,mg\,m^{-2}$); ■ = asparginase $1{,}000\,IU\,kg^{-1}\,day^{-1}$ i.v.; ▥ = asparginase $5{,}000\,IU\,kg^{-1}\,day^{-1}$ i.v.; ASP = $10{,}000\,IU\,kg^{-1}\,day^{-1}$ i.v.; CYC = cyclophosphamide ($1\,g\,m^{-2}$ i.v.); Ⓒ = cytosar-arabinoside ($75\,mg\,m^{-2}$ i.v. daily for 4 days); 6MP = oral 6-mercaptopurine ($75\,mg\ m^{-2}\,day^{-1}$); MTX = oral methotrexate ($20\,mg\,m^{-2}\,week^{-1}$); M = intrathecal methotrexate ($12\,mg\,m^{-2}$); pred/p = oral prednisone ($60\,mg\,m^{-2}\,day^{-1}$); —— = 6-thioguanine ($60\,mg\,m^{-2}\,day^{-1}$); ▧ / ▩ = 500/1,000 mg MTX 24 h infusion with leucovorin rescue; V = vincristine ($2\,mg\,m^{-2}$, max. $2\,mg\,dose^{-1}$); XXXX = CNS-irradiation (2,400 cGy).

### Patients and methods

### *Patients*

From July 1981 to December 1985, >90% of all new Danish cases of non-B-cell ALL ⩾1 and ⩽15 years of age were treated by common therapy programmes (Figure 1). One hundred and twenty-eight patients completed induction and consolidation therapy. Of these, 13 were excluded from this study due to major protocol violation during induction or consolidation therapy, refusal of MT by parents, AT not measured during MT, or lack of data (3, 1, 7 and 2 patients). Thus, 115 patients (67 boys and 48 girls) were eligible for analyses, with 56 cases of standard risk (SR), 39 cases of intermediate risk (IR) and 20 cases of high risk ALL. Criteria for risk classification are given in Table I.

Duration of remission is the number of days between compete clinical remission and end of follow-up (1 November 1988), or first positive manifestation of relapse (>5% blast in bone-marrow, or any extramedullary, histologically confirmed leukaemia). For patients still in remission, median length of follow-up from achieved remission was 60 months (range: 34–87 months). No patients in continuous complete remission (CCR) died or were lost for follow-up. One patient with HR-ALL had a bone-marrow transplantation in first remission during the first year of MT and was censored at this event (Simon & Wittes, 1985).

### *Therapy*

Therapy depended on risk classification (Figure 1). MT with weekly oral MTX (target dose: $20\,mg\,m^{-2}$ ) and daily oral

Correspondence: K. Schmiegelow.
Received 28 July 1989; and in revised form 3 December 1989.

**Table I** Criteria for risk-classification

| | |
|---|---|
| Standard risk | 1. WBC $\leqslant 20 \times 10^9 l^{-1}$ |
| | 2. Age $\geqslant 2$ and $< 10$ years |
| | 3. No CNS-leukaemia or mediastinal mass |
| | 4. Non-T-cell ALL |
| Intermediate risk | 1. WBC $\geqslant 20$ and $< 50 \times 10^9 l^{-1}$ |
| | 2. Age $< 2$ or $\geqslant 10$ years, and WBC $< 50 \times 10^9 l^{-1}$ |
| | 3. No CNS-leukaemia or mediastinal mass |
| | 4. Non-T-cell ALL |
| High risk | One or more of the following: |
| | 1. WBC $\geqslant 50 \times 10^9 l^{-1}$ |
| | 2. CNS-leukaemia |
| | 3. Mediastinal mass |
| | 4. T-cell ALL |

Patients with B-cell ALL and patients $< 1$ year at diagnosis are not included.

6MP (target dose: $75 \text{ mg m}^{-2}$) was started 10–12 weeks from remission with dose adjustments determined by blood counts (target WBC: $1.5–4.0 \times 10^9 l^{-1}$) and presence of toxicity. Drug dosage was reduced for WBC $< 1.5 \times 10^9 l^{-1}$, both drugs being withdrawn at WBC $< 1.0 \times 10^9 l^{-1}$. All patients with IR-ALL received 3–5 cycles of alternating therapy of reinductions or 24 h intravenous high dose MTX-infusions (HDM) at 4-week intervals during the first 6–9 months of MT. In addition, patients with IR-ALL diagnosed before 1983 (11 patients) and all patients with HR-ALL received reinductions every third month throughout MT. Therapy was for all patients discontinued after three years of CCR.

During MT and for at least 12 months after cessation of therapy, haemoglobin, white cell (WBC) and platelet counts were done at least monthly. Routine absolute neutrophile counts (ANC) were not part of the protocols. ANC were determined together with WBC for 59 patients. AT was measured regularly during MT, but the intervals between sampling differed. AT was for 17 patients measured together with blood counts; i.e. at least monthly. For 63 patients, AT was measured every 6–8 weeks, and for the remaining 25 patients at longer intervals (median interval: 4 months). AT was determined as described elsewhere (Scandinavian Committee on Clinical Chemistry, 1974). Bone-marrow and spinal fluid tests were done before the cessation of therapy, every third month for the first year off treatment, and beyond this only at suspicion of relapse. Cases of haematological relapse include relapses in combination with extramedullary disease. CNS (central nervous system) and testicular relapses are all isolated relapses, and are counted as censoring observations in respect to haematological remission (Simon & Wittes, 1985).

### *Drug dosage*

For each patient, the average dose of MTX and 6MP per $m^2$ were calculated as the cumulated prescribed dose per $m^2$ divided by the period from start of MT until relapse, cessation of therapy or end of follow-up, whichever came first. The duration of withdrawal of MTX and 6MP during MT due to toxicity or febrile illness were calculated as a percentage of the length of MT.

### *WBC*

For every patient, a mean WBC was calculated for the period of MT until relapse, end of therapy or end of follow-up ($mWBC_{MT}$). For patients completing MT, a mean WBC was calculated for the third year of MT ($mWBC_{3y}$). They were calculated as weighted means of all WBC measurements: $mWBC_{MT} = (\Sigma_F^L WBC_n \times (D_{n+1} - D_n))/L_{MT}$, $mWBC_{3y} = (\Sigma_F^L WBC_n \times (D_{n+1} - D_n))/365$, where $WBC_n$ is the white cell count at date n, $D_F$ and $D_L$ are the first and the last days of the period included in the analyses, and $L_{MT}$ is the length of MT. Thus, each WBC was weighted according to the intervals between sampling. For patients being at least 10 months off therapy in first remission, a mean WBC after cessation of therapy ($mWBC_{off}$) was similarly calculated for a period delimited by the date after which the intervals between WBC measurements exceeded 10 weeks (median length: 19 months, 10–33). Calculations of $mWBC_{off}$ included every white cell count measured from one month off therapy and throughout the period. The rise in mWBC following cessation of therapy was calculated as $mWBC_{off} - mWBC_{3y}$. Similarly a $mANC_{MT}$ and the rise in mANC were calculated for the 59 patients with regular ANC determinations.

### *Hepatotoxicity*

For each patient, the degree of hepatotoxicity was calculated as a weighted mean of all AT determinations available during each year of MT ($mAT_{1y}$, $mAT_{2y}$, $mAT_{3y}$) excluding those within 3 weeks after HDM. mAT were calculated as: $mAT = (\Sigma_F^L P_n \times AT_n)/\Sigma_F P_n$, where $P_n = (D_{n+1} - D_n)$, $D_{n+1}$ is the first date to follow with blood counts, whether or not it includes measuring of AT, $D_F$ is the first and $D_L$ the last date with AT measurements of the year in question $AT_n$ is the AT value on date $D_n$. Similarly, a mean AT was calculated for the whole period of MT ($mAT_{MT}$). Patients were defined as not having hepatotoxicity, if $mAT_{MT}$ was $\leqslant 40 \text{ IU } l^{-1}$.

Due to persistent, severe hepatotoxicity, oral MTX was in 13 patients substituted with oral cyclophosphamide ($100 \text{ mg m}^{-2} \text{ day}^{-1}$). These patients were all censored at the time of cyclophosphamide/MTX subsititution (Simon & Wittes, 1985).

### *Statistical analyses*

Cox proportional hazards regression analysis with maximum partial likelihood ratio tests was applied to a set of variables to detect possible prognostic factors as well as the combined and independent significance of these (Cox, 1972). In the stepwise multivariate analysis, additional parameters were included in the combined model only if their level of significance was $< 0.10$. The Kaplan–Meier method was applied to estimate remission duration and to generate relapse-free survival curves (Kaplan & Meier, 1958). Remission duration of subgroups were compared with the generalised Savage test (Mantel, 1966).

### Results

The median $mAT_{MT}$ was $48 \text{ IU } l^{-1}$ (range: 17–287) (Figure 2). Forty patients had a $mAT_{MT} \leqslant 40 \text{ IU } l^{-1}$. Nine of these 40 patients never had an AT of $> 40 \text{ IU } l^{-1}$ during MT, whereas intermittent rises in AT of $> 40 \text{ IU } l^{-1}$ were found in the remaining 31 patients.

For the total material there was no significant differences

**Figure 2** Histogram of mean alanine aminotransferases ($mAT_{MT}$) ($IU\ l^{-1}$) during maintenance therapy (115 patients).

in the prevalence of hepatotoxicity during the first, second, and third year of MT (median values for $mAT_{1y} = 53$, $mAT_{2y} = 50$, $mAT_{3y} = 49$ IU $l^{-1}$). Neither risk group (which includes previous therapy), gender, or age significantly influenced $mAT_{MT}$, and there was no significant relation between $mAT_{MT}$ and the interval between samplings, although there was a trend towards patients with hepatotoxicity having AT measured at shorter intervals than patients with normal AT.

A highly significant intra-individual correlation between the degree of hepatotoxicity during the first, second, and third year of MT could be demonstrated (Figure 3).

Patients with $mAT_{MT} > 40$ IU $l^{-1}$ did not differ significantly from patients with $mAT_{MT} \leqslant 40$ IU $l^{-1}$ in respect to gender, age at diagnosis, risk group (which includes previous therapy), mean dose of MTX and 6MP $m^{-2}$, or $mWBC_{MT}$ (Table II).

All patients in CCR with a $mAT_{MT} > 80$ IU $l^{-1}$, had normal AT ($<40$ IU $l^{-1}$) by one year off MT.

A liver biopsy was obtained from a 10-year-old girl, who had a 10–20-fold rise in AT, a 2–3-fold rise in serum bilirubin, normal prothrombin time, and negative serology for $HB_SAg$. The histology was normal. No other patients had a biopsy.

*Drug dosage*

There was a significant relation between $mAT_{MT}$ and the percentual period of withdrawal of MTX ($r = 0.34$, $P < 0.0001$) and 6MP ($r = 0.25$, $P = 0.003$) during MT. When patients with withdrawal of MTX or 6MP for more than 10% of the length of MT were excluded, no significant relation between $mAT_{MT}$ and the dose of MTX and 6MP could be found (MTX: $r = -0.07$, $P = 0.34$; 6MP: $r = -0.15$, $P = 0.11$). The mean period of withdrawal of MTX and/or 6MP (as previously defined) was 55% longer for relapse patients with $mAT_{MT} > 40$ IU $l^{-1}$ compared to relapse patients with $mAT_{MT} \leqslant 40$ IU $l^{-1}$ ($P < 0.001$) (Wilcoxon's rank sum test). The major reason for drug-withdrawal for $> 10\%$ of MT was hepatotoxicity.

*Myelotoxicity*

No significant relation could be demonstrated between $mAT_{MT}$ and $mWBC_{MT}$ ($r = -0.03$, $P = 0.36$). However, a weak correlation existed between $mAT_{3y}$ and the rise in white cell count following cessation of therapy ($r = 0.24$, $P = 0.06$). A similar relation was found between $mAT_{MT}$ and $mANC_{MT}$. However, it was not significant possibly due to the low number of patients.

**Figure 3** Scattergram of the relation of mean alanine aminotransferase (IU $l^{-1}$) during the first and second, and during the second and third year of maintenance therapy (MT), respectively. First *vs* second year of MT: $r = 0.72$, $P < 0.00001$, intercept = 15.5, slope = 0.66; second *vs* third year of MT: $r = 0.79$, $P < 0.00001$, intercept = 27.8, slope = 0.70.

*Relapse rate*

A total of 38 patients relapsed during follow-up, i.e. 24 haematological, eight CNS and six testicular relapses. Boys had an increased relapse risk compared to girls ($P = 0.003$), but did not differ in respect to duration of haematological remission. Four and a half year probability of CCR did not differ among the risk groups (SR, 0.62,; IR, 0.57; HR, 0.68); neither did probability of haematological remission (SR, 0.71; IR, 0.74; HR, 0.81).

**Table II** Characteristics of patients without or with hepatotoxicity[a]

| | *Total* | $mAT_{MT}$[b] $<40$ *IU* $l^{-1}$ | $mAT_{MT} > 40$ *IU* $l^{-1}$ |
|---|---|---|---|
| No. patients Male:female | 67:48 | 25:21 | 42:27 |
| Mean age ± 1 s.d. | 5y11m ± 3y8m | 5y8m ± 3y10m | 6y2m ± 3y7m |
| Risk groups HR/IR/SR | 56/39/20 | 21/19/6 | 35/20/14 |
| Relapses BM/CNS/TESTES | 24/8/6 | 15/4/2 | 9/4/4 |
| Mean WBC[c] | 4.2 ± 1.1 | 4.3 ± 1.2 | 4.1 ± 0.9 |
| Mean dose of MTX[d] | 15.4 ± 4.1 | 15.8 ± 4.7 | 15.1 ± 3.6 |
| Mean dose of 6MP[d] | 63.7 ± 10.1 | 65.9 ± 12.0 | 62.2 ± 8.3 |
| Withdrawal of MTX[e] for relapse patients | | | |
| < 5% of the length of MT | | 13 (62%) | 7 (41%) |
| ⩾ 5 and < 10% | | 3 (14%) | 4 (24%) |
| ⩾ 10% | | 5 (24%) | 6 (35%) |
| Withdrawal of 6MP[e] for relapse patients | | | |
| < 5% of the length of MT | | 14 (67%) | 8 (47%) |
| ⩾ 5 and < 10% | | 5 (24%) | 2 (12%) |
| ⩾ 10% | | 2 ( 9%) | 7 (41%) |

[a]Defined as $mAT_{MT} \leqslant$ or $> 40$ IU $l^{-1}$. [b]Mean alanine aminotransferase during maintenance therapy; [c] $\times 10^9$ $l^{-1}$; [d]mg $m^{-2}$; [e]Numbers and fractions of relapse-patients with different degrees of drug-withdrawal due to toxicity or febrile illness.

Patients who relapsed had significantly lower $mAT_{MT}$ than patients staying in remission (Wilcoxon's rank sum test: any relapse, $P = 0.03$; haematological relapse, $P = 0.03$).

Stepwise multivariate regression analyses were done to explore the prognostic significance of combinations of possible relapse predictors and to define a 'best-fit' model to predict relapse. The variables analysed were: year of diagnosis (1981–83 = 1 *vs* 1984–85 = 2), gender (male = 1, female = 2), age at diagnosis, WBC at diagnosis, the average dose of MTX and 6MP, $mWBC_{MT}$, and presence of hepatotoxicity ($mAT_{MT} \leqslant 40$ I $l^{-1}$ = 1, $mAT_{MT} > 40$ IU $l^{-1}$ = 2). The best-fit model to predict haematological relapse included hepatotoxicity and $mWBC_{MT}$ (global $P$ value $< 0.02$). The best-fit model to predict any relapse included gender and hepatotoxicity (global $P$ value $< 0.002$). The coefficients of the covariates included in the models and their $P$ values as uni- and covariates are given in Table III. Identical 'best-fit' models were achieved with stepwise backward elimination, all covariates being forced into the model at the first step.

Figure 4 illustrates the positive relation between $mAT_{MT}$ and the probability of staying in remission. Figure 5 gives Kaplan–Meier plots for the risk of haematological or any relapse for patients stratified by a $mAT_{MT} > 40$ IU $l^{-1}$ or $\leqslant 40$ IU $l^{-1}$. Patients with a $mAT_{MT} \leqslant 40$ IU $l^{-1}$ had a significantly poorer outcome than did patients with $mAT_{MT} > 40$ IU $l^{-1}$ with a 4.5 year probability of staying in haematological remission of 0.63 and 0.83 ($P = 0.03$), and a 4.5 year probability of CCR of 0.50 and 0.70 ($P = 0.06$). The favourable prognosis for patients with a $mAT_{MT} > 40$ IU $l^{-1}$ existed for all three risk groups, but was statistically significant only in respect to haematological relapse for SR patients (Table IV). A time-dependency of the prognostic value of hepatotoxicity (i.e. a difference in the clinical significance of hepatotoxicity during the first, second and third years of MT) could not be demonstrated.

**Figure 4** Probability of staying in complete or hematological remission for subgroups of patients delimited by their mean alanine aminotransferase. No. of patients in each group are given on top of columns. ▧ CCR, ▨ Hematol. remission

### Discussion

The present study has confirmed the results of previous reports of a high prevalence of hepatotoxicity in leukaemic children receiving oral MTX/6MP therapy. Whereas a number of these studies have set focus on the relation between pathological liver function tests and the presence of an abnormal liver histology (Nesbit *et al.*, 1976; McIntosh *et al.*, 1977; Topley *et al.*, 1979), the main purpose of the present study was to explore the relation between relapse risk and the rise in serum AT, the most commonly applied test to detect liver dysfunction used by the departments participating in the study.

The significant correlation between a rise in serum AT and a reduced relapse risk could have several explanations, alone or combined.

The large inter-individual differences in plasma concentration profiles after oral MT (Poplack *et al.*, 1986) implies different intensity of therapy, and children with adverse pharmacokinetic parameters might lack hepatotoxicity and be at increased risk for relapse, due to reduced systemic drug exposure to both liver and lymphoblasts (Pinkel *et al.*, 1971; Craft *et al.*, 1981). In support of hepatotoxicity reflecting treatment intensity, we found a relation between $mAT_{MT}$ and the rise in white cell counts following cessation of therapy. In addition, a correlation of hepatotoxicity to red blood cell and hepatic accumulation of MTX-polyglutamates has been demonstrated for MTX-therapy of leukaemia and psoriasis (Schmeigelow *et al.*, 1989; Hendel, 1985). Finally, patients with hepatoxicity seem to have delayed systemic clearance of MTX (Parker *et al.*, 1980). If hepatotoxicity does reflect treatment intensity, reduction of the doses of MTX and 6MP for patients with abnormal liver function tests could carry an increased relapse risk (Pinkel *et al.*, 1971), which was also indicated by the present study.

6MP undergoes hepatic metabolism and the first-pass effect is considerable (Zimm *et al.*, 1983). MTX-induced hepatotoxicity, whether or not due to increased intestinal absorption or reduced clearance, could therefore impair 6MP breakdown, leading to treatment intensification. Along this line it has been demonstrated that 6MP plasma peak-concentration and AUC are increased with concurrent administration of MTX and 6MP, compared to 6MP given alone (Balis *et al.*, 1987).

Another metabolic explanation could be changes in the activity of enzymes involved in MTX and 6MP metabolism (Zimm *et al.*, 1986; Lennard & Lilleyman, 1987; Rodenhuis *et al.*, 1987). Zimm *et al.* (1986) demonstrated significant changes in lymphoblastic activity of enzymes involved in 6MP metabolism, when malignant lymphoblasts at diagnosis and relapse were compared. It is not known whether a parallel exists between such changes in the lymphoblastic clone and the liver, in which case reduced hepatotoxicity could parallel an increased risk for relapse. If such a correlation exists, tapering of hepatotoxicity before the event of relapse would be expected, but we found no such correlation.

Drug compliance cannot be excluded as a possible contributing factor to both a low $mAT_{MT}$ and an increased risk of relapse. However, a recent study indicated compliance to be high among Danish leukaemic children (Schmiegelow *et al.*, 1990).

The relation between hepatotoxicity and relapse risk could be due to a prolonged effect of previously given hepatotoxic drugs, which could have influenced the amount of residual

**Table III** Stepwise multivariate proportional hazards regression models

| | *Best-fit models for* | | | |
|---|---|---|---|---|
| | *Haematological relapse risk Global P value* $<0.02$ | | *Overall relapse risk Global P value* $<0.002$ | |
| *Covariates* | *Step 0* | *Step 1* | *Step 0* | *Step 1* |
| Gender[a] | 0.16 | 0.11 | 0.003[c] | 0.003 (−1.13) |
| Mean WBC during therapy | 0.06 | 0.09[c] (0.28) | 0.27 | 0.37 |
| Hepatotoxicity[b] | 0.03[c] | 0.03 (−0.82) | 0.06 | 0.03[c] (0.70) |

[a]Male = 1, female = 2. [b]Mean AT $\leqslant 40$ IU $l^{-1}$ = 1, mean AT $> 40$ IU $l^{-1}$ = 2. [c]Variable included in the model at the following step. Values are $P$ values.

**Figure 5** Kaplan–Meier curves for probability of haematological or complete continuous remission for patients defined by the mean alanine aminotransferase during maintenance therapy ($mAT_{MT}$): $mAT_{MT}$ $\leqslant 40\ IU\ l^{-1}$ (lower curves), $mAT_{MT}$ $>40\ IU\ l^{-1}$ (upper curves). $P=0.03$ and $P=0.06$, respectively, for differences in hematological and complete remission. Patients at risk: $mAT_{MT}$ $<40\ IU\ l^{-1}$: 1 year from remission, 43; 3 years, 28; 5 years, 12; $mAT_{MT}$ $>40\ IU\ l^{-1}$: 1 year, 62; 3 years, 42; 5 years, 17.

disease as well as liver tolerance to the hepatotoxic effect of MTX and 6MP. But the finding of a correlation between relapse risk and the degree of hepatotoxicity for all risk groups, and in addition, this being statistically significant for SR-patients who received only vincristine, prednisone and HDM during induction and consolidation therapy, make this explanation unlikely.

Lack of doctor compliance has been suggested as a possible risk factor (Peeters *et al.*, 1988). In the present material we found no difference in the dose of MTX for patients with $mAT_{MT}$ $\leqslant 40$ or $>40\ IU\ l^{-1}$. Neither did the two groups

**Table IV** Probability of haematological or complete remission for patients stratified by risk group and mean serum AT during maintenance chemotherapy

| | *Risk groups* | | |
|---|---|---|---|
| | *SR* | *IR* | *HR* |
| No. of patients with $mAT_{MT} \leqslant 40$ *vs* $> 40\ IU\ l^{-1}$ | 21/35 | 19/20 | 6/14 |
| Probability of | | | |
| haematological remission[a] | 0.57/0.80[b] | 0.68/0.81 | 0.62/0.91 |
| complete remission[a] | 0.52/0.68 | 0.48/0.70 | 0.50/0.77 |

AT, alanine aminotransferase; $mAT_{MT}$, mean AT during maintenance chemotherapy; SR/IR/HR, standard/intermediate/high risk. [a]Estimates at 4.5 year from achieved remission. [b]$P<0.05$.

differ in respect to the degree of leukopenia ($mWBC_{MT}$) the parameter most widely used to adjust MT. Thus, there was no indication that patients lacking hepatotoxicity were treated too leniently.

Other potentially hepatotoxic events like previous given drugs, general anaesthesia (GA) or hepatitis could have influenced the prevalence of rises in AT. However, as therapy and the frequency of GA were the same for all patients within the risk groups, and AT was the only abnormal liver function test (among prothrombin time, bilirubin, phosphatase and AT) for >90% of the patients with $mAT_{MT}$ $>40\ IU\ l^{-1}$, these factors hardly significantly influenced the relation between rises in AT and risk of relapse.

A major question is whether downward dose adjustments are justified in the case of hepatotoxicity. A number of previous reports have indicated a high incidence of serious liver damage, but most of these patients received far higher doses than presently used (Parker *et al.*, 1980). The rises in liver enzymes during MT are in most cases mild and transient with normalisation within a few months following the cessation of therapy. For patients with severe liver dysfunction with affected prothrombin time, jaundice or very high levels of liver enzymes, a liver biopsy may offer a guide to whether dose modifications should take place, though often no correlation exists between pathological liver function tests and histology (Topley *et al.*, 1979). Parker *et al.* (1980) suggested that analysing plasma concentration profiles of drugs could be helpful in determining dose modifications. However, one should bear in mind that patients with delayed drug clearance might be receiving the better treatment, which could be jeopardised by dose reductions.

Until prospective studies have demonstrated that hepatotoxicity is a greater threat to patients than the risk of relapse, dose reductions do not seem warranted unless serious liver damage have been confirmed by histology.

This study has received financial support from The Jenny and Mogens Vissing Foundation, The Rosalie Petersen Foundation and Inner Wheel Denmark. The authors thank the following medical and paediatric departments for making their records available to them: Glostrup Hospital and Hvidovre Hospital, Copenhagen; University Hospital, Odense; Ålborg Hospital, Ålborg; University Hospital, Århus.

### References

BALIS, F.M., HOLCENBERG, J.S., ZIMM, S. & 6 others (1987). The effect of methotrexate on the bioavailability of oral 6-mercaptopurine. *Clin. Pharmacol. Ther.*, **41**, 384.

COX, D.R. (1972). Regression models and life-tables. *J. R. Stat. Soc. B*, **34**, 187.

CRAFT, A.W., RANKIN, A. & AHERNE, W. (1981). Methotrexate absorption in children with acute lymphoblastic leukemia. *Cancer Treat. Rep.*, **65**, suppl. 1, 77.

HARB, J.M., KAMEN, B.A., WERLIN, S.L., BLANK, E.L., CAMITTA, B.M. & OECHLER, H. (1983). Hepatic ultrastructure in leukemic children treated with methotrexate and 6-mercaptopurine. *Am. J. Pediatr. Hematol. Oncol.*, **5**, 323.

HENDEL, J. (1985). Clinical pharmacokinetics of methotrexate in psoriasis therapy. *Dan. Med. Bull.*, **32**, 329.

KAPLAN, E.L. & MEIER, P. (1958). Non-parametric estimation from incomplete observations. *J. Am. Stat. Assoc.*, **53**, 457.

LENNARD, L., REES, C.A., LILLEYMAN, J.S. & MADDOCKS, J.L. (1983). Childhood leukemia: a relationship between intracellular 6-mercaptopurine metabolites and neutropenia. *Br. J. Pharmacol.*, **16**, 359.

LENNARD, L. & LILLEYMAN, J.S. (1987). Are children with acute lymphoblastic leukemia given enough mercaptopurine? *Lancet*, **ii**, 785.

MCINTOSH, S., DAVIDSON, D.L., O'BRIEN, R.T. & PEARSON, H.A. (1977). Methotrexate hepatotoxicity in children with leukemia. *J. Pediatr.*, **90**, 1019.

MANTEL, N. (1966). Evaluation of survival data and two new rank order statistics arising in its consideration. *Cancer Chemother.*, **50**, 163.

MENARD, D.B., GISSELBRECHT, C., MARTY, M., REYE, F. & DHUMEAUX, D. (1980). Antineoplastic agents and the liver. *Gastroenterology*, **78**, 142.

NESBIT, M., KRIVIT, W., HEYN, R. & SHARP, H. (1976). Acute and chronic effects of methotrexate on hepatic, pulmonary and skeleton system. *Cancer*, **37**, 1048.

PARKER, D., BATE, C.M., CRAFT, A.W., GRAHAM-POLE, J., MALPAS, J.S. & STANSFELD, A.G. (1980). Liver damage in children with acute leukemia and non-Hodgkin's lymphoma on oral maintenance chemotherapy. *Cancer Chemother. Pharmacol.*, **4**, 121.

PEETERS, M., KOREN, G., JAKUBOVICZ, D. & ZIPURSKY, A. (1988). Physician compliance and relapse rates of acute lymphoblastic leukemia in children. *Clin. Pharmacol. Ther.*, **43**, 228.

PINKEL, D., HERNANDEZ, K., BORELLA, L. & 4 others (1971). Drug dosage and remission duration in childhood lymphocytic leukemia. *Cancer*, **37**, 47.

POPLACK, D.G., BALIS, F.M. & ZIMM, S. (1986). The pharmacology of orally administered chemotherapy. *Cancer*, **58**, 473.

RODENHUIS, S., KREMER, J.M. & BERTINO, J.R. (1987). Increase in dihydrofolate reductase in peripheral blood lymphocytes of rheumatoid arthritis patients treated with low-dose oral methotrexate. *Arthr. Rheum.*, **30**, 369.

SCANDINAVIAN COMMITTEE ON CLINICAL CHEMISTRY (1974). Recommended methods for the determination of four enzymes in blood. *Scand. J. Clin. Lab. Invest.*, **32**, 291.

SCHMIEGELOW, K., SCHRØDER, H., PULCZYNSKA, M.K. & HEJL, M. (1990). Maintenance chemotherapy for childhood acute lymphoblastic leukemia: relation of bone-marrow- and hepatotoxicity to the concentration of methotrexate in erythrocytes. *Cancer Chemother. Pharmacol.* (in the press).

SIMON, R. & WITTES, R.E. (1985). Methodological guidelines for reports of clinical trials. *Cancer Treat. Rep.*, **69**, 1.

TOPLEY, J.M., BENSON, J., SQUIER, M.V. & CHESSELS, J.M. (1979). Hepatotoxicity in the treatment of acute lymphoblastic leukemia. *Med. Pediatr. Oncol.*, **7**, 393.

ZIMM, S., COLLINS, J.M., O'NEILL, D., CHABNER, B.A. & POPLACK, D.G. (1983). Inhibition of first-pass metabolism in cancer chemotherapy: interaction of 6-mercaptopurine and allopurinol. *Clin. Pharmacol. Ther.*, **34**, 810.

ZIMM, S., REAMAN, G., MURPHY, R.F. & POPLACK, D.G. (1986). Biochemical parameters of 6-mercaptopurine in patients with acute lymphoblastic leukemia. *Cancer Res.*, **46**, 1495.